# Expression of Repressor Element 1 Silencing Transcription Factor (REST) in Serotonin Neurons in the Adult Male Nile Tilapia (*Oreochromis niloticus*)

**DOI:** 10.3389/fnana.2020.599540

**Published:** 2021-03-11

**Authors:** Tomoko Soga, Shingo Nakajima, Ishwar S. Parhar

**Affiliations:** Brain Research Institute Monash Sunway (BRIMS), Jeffery Cheah School of Medicine and Health Sciences, Monash University Malaysia, Selangor, Malaysia

**Keywords:** REST, neuron-retrictive silencer factor, serotonin neuron, hindbrain, midbrain area

## Abstract

Repressor element-1 silencing transcription factor (REST) is highly expressed in the dorsal raphe where serotonin (5-hydroxytryptamine, 5-HT) neurons are located. REST works as a transcription factor for the 5-HT receptor and tryptophan hydroxylase two-gene expression. We hypothesized that REST is co-expressed in 5-HT neurons, which, if demonstrated, would be useful to understand the mechanism of 5-HT dysfunction-related disorders such as negative emotions and depression. Therefore, the present study was designed to examine the expression of the *REST* gene in the brain (forebrain, midbrain, and hindbrain) of adult male Nile tilapia (*Oreochromis niloticus*) using rt-PCR. Besides, using immunocytochemistry, co-localization of the *REST* gene was examined in 5-HT neurons and with neuronal-/glial-cell markers. We found a high expression of the *REST* gene in the midbrain region of the dorsal raphe, an area of 5-HT neurons. Double-label immunocytochemistry showed neuron-specific expression of *REST* co-localized in 5-HT neurons in the dorsal and ventral parts of the periventricular pretectal nucleus, paraventricular organ, and dorsal and medial raphe nucleus. Since midbrain 5-HT neurons express REST, we speculate that REST may control 5-HT neuronal activity related to negative emotions, including depression.

## Introduction

Repressor element 1 silencing transcription factor (REST), also known as neuron-restrictive silencing factor (NRSF), shows gene silencing transcription activities of target genes, which contain the repressor element-1 (RE-1) binding site (Calderone et al., 2003; Bruce et al., 2004; Schiffer et al., 2014). In neurons, REST regulates the transcription of hundreds of neuronal genes, including genes that encode for neurotransmitter receptors, transporters, neurotrophic receptors, and genes that encode for proteins involved in vesicular function, axonal guidance, and ion channels (Bruce et al., 2004). Studies have implicated changes in the expression of REST, as well as REST-dependent genes, in the specific brain regions and several brain diseases, including Alzheimer’s disease, Huntington disease, Parkinson’s disease, ischemia, epilepsy, and depression (Goswami et al., 2010; Baldelli and Meldolesi, 2015).

Previous studies have indicated that REST affords neuroprotective function in several cellular signaling pathways, leading to neuronal cell survival (Song et al., 2017). On the other hand, reduced nuclear *REST* gene expression in the aging human brain and patients with Alzheimer’s disease (Lu et al., 2014; Meyer et al., 2019) induces cell death-related genes and loss of neurons, which leads to cognitive dysfunction and neurodegenerative disease (Hwang and Zukin, 2018).

There is growing evidence that alteration of *REST* gene expression is one of the key components of stress and the pathophysiology of depression, aberrant *REST*, and *REST*-associated genes (Otsuki et al., 2010; Soga et al., 2020). Clinical study reports female depressed patients to show higher expression of *REST* gene in the dorsal raphe neurons (Goswami et al., 2010). In the serotonin (5-hydroxytryptamine, 5-HT) system, REST inhibitory effect regulates 5-HT_1A_ receptor gene expression by binding to the RE-1 site in the 5-HT_1A_ promoter region (Lemonde et al., 2004). Thus, increased *REST* mRNA levels suppress 5-HT_1A_ expression in the dorsal raphe of patients who suffer from depression (Goswami et al., 2010). Besides, REST regulates the promoter activity of the tryptophan hydroxylase-2 (TPH2) gene in raphe 5-HT biosynthesis (Patel et al., 2007; Gentile et al., 2012; Nawa et al., 2017). Although the cellular localization of REST in neurons and astrocytes has been reported (Abrajano et al., 2009a, b; Prada et al., 2011; Pajarillo et al., 2020), the expression of REST in 5-HT neurons and their cellular function in 5-HT related mental disorders remain poorly understood.

The Nile tilapia, *Oreochromis niloticus*, is an emerging model for social neuroscience due to its well-characterized behaviors, physiology, and neuroendocrine systems (Meek, 1998; Uchida et al., 2005; Higuchi et al., 2018; Lim et al., 2020). In particular, social behavior in the Nile tilapia is well studied; the change in body color, relative to social rank, triggered by dynamic social interactions is an excellent feature in understanding brain chemistry changes in different social statuses (Ogawa et al., 2006; Lim et al., 2020). In tilapia brain, there are three 5-HT neuronal populations: the paraventricular organ (PVO), the dorsal and ventral periventricular pretectal nuclei (PPd and PPv), and the superior and inferior raphe (SR and IR; Cham et al., 2018; Higuchi et al., 2018).

In this study, we investigated the expression of the *REST* gene in 5-HT neurons. We first determined the *REST* gene expression pattern in micro-dissected brain regions (forebrain, midbrain, and hindbrain) of the male Nile tilapia by rt-PCR. Next, we studied whether the *REST* gene is expressed in neurons or glial cells. Finally, *REST*-positive cells were localized in the midbrain and co-localized with 5-HT neurons using double-label *in situ* hybridization (ISH) and immunohistochemistry (ICC). The identification of REST in 5-HT neurons will serve as an initial step for our future study to understand the involvement of serotonergic regulation of negative emotions such as depression and fear.

## Materials and Methods

### Animals

Sexually matured male Nile tilapia fish (*Oreochromis niloticus*) were used in this experiment (*n* = 11). All animals were kept in standard fish tanks (size: H90 × W25 × D25 cm) with 10–20 fish per tank under standard housing conditions: lighting (14-h light: 10-h dark cycle), freshwater aquaria maintained at 28 ± 0.5°C, equipped with circulating water system, and given aeration regularly. Cichlid food pellets [Star feed TP-1, Star Feedmis (M) Sdn Bhd] were given thrice a day. Samples were used for histological studies such as ISH (*n* = 6) and double ISH and ICC (*n* = 5). All experimental procedures were approved and conducted according to the guidelines of Monash University Animal Ethics Committee, AEC (approval: MARP/2015/109, MARP/2015/180).

### Gene Expression Study

#### Preparation of Brain Samples

Adult male fish were anesthetized with 0.02% benzocaine (Sigma–Aldrich, St. Louis, MO, USA), and the brains were collected and immediately frozen in Tissue-Tek^®^ O.C.T. Compound (Sakura Finetek USA, Inc., Torrance, CA, USA) for brain microdissection, then stored at −80°C until use. Microdissected brain samples were prepared using a cryostat (Cham et al., 2017). Briefly, frozen coronal sections (60 μm) were taken from the cryostat (Leica CM1860) followed by mounting onto microscope slides (Sail Boat Lab Company, Zhejiang, China). Tissues were collected by a 200-μl pipette tip and immersed into TRIzol (Thermo Fisher Scientific, Waltham, MA, USA). Based on our previous studies, brain regions were targeted and collected as follows: telencephalon (~55 sections), pre-optic area (~20 sections), optic tectum (~65 sections), midbrain (~10 sections), hypothalamus (~50 sections), cerebellum (~80 sections), and hindbrain (~85 sections; Cham et al., 2017).

#### RNA Extraction and cDNA Synthesis

RNA extraction and cDNA preparation were performed according to Higuchi et al. (2018). Briefly, each brain sample was homogenized with 200 μl TRIzol to extract total RNA. Forty microliter of chloroform was added at 1:5 of the original volume of TRIzol. Then, the tube was incubated for 3 min at room temperature (RT) followed by the centrifugation for 15 min at 12,000 *g* at 4°C. To precipitate RNA, only the colorless supernatant was moved to a new tube and admixed with 100 μl of isopropyl alcohol. After the incubation for 10 min at RT, the tube was centrifuged for 15 min at 12,000 *g* at 4°C. At the final step, the RNA pellet washing step was done twice with 75% ethanol and resolved in 20 μl Ultrapure Milli-Q water after drying. Each RNA sample (1,000 ng/μl) was translated into cDNA with High capacity cDNA Reverse Transcription Kit (Applied Biosystems, Foster City, CA, USA), following the manufacturer’s protocol. The PCR conditions for all reactions were as follows: for 10 min at 25°C; for 120 min at 37°C, and for 5 min at 85°C. The cDNA was kept at −20°C until use.

### Real-Time PCR

The quantification of *REST* and *β-actin* mRNA was performed using forward and reverse primers according to the GenBank sequence (Table 1). Both genes were cloned in the pGEM-T Easy Vector plasmid (Promega, Madison, WI, USA). After cloning, the sequencing of the cloned Nile tilapia *REST* (92 bp) and *β-actin* (120 bp) fragment was conducted with BigDye Terminator v3.1 Cycle Sequencing kit (Applied Biosystems, Foster City, CA, USA) in 3310 Genetic Analyzer (Applied Biosystems, Foster City, CA, USA). The PCR was carried out using SensiFAST SYBR Hi-ROX Kit (Bioline, Taunton, MA, USA), and the PCR mixture (10 μl) contained 0.2 μM primers and 1 μl cDNA. The PCR conditions for all reactions were as follows: for 2 min at 95°C; 40 cycles for 5 s at 95°C and 30 s at 60°C, and a final dissociation step for melting curve analysis. The results were analyzed by the ΔΔCt method using *β-actin* as the reference gene. The absolute copy number of *REST* mRNA was also determined. The plasmid comprising the *REST* gene was diluted continuously to concentrations of 10^9^, 10^8^, 10^7^, 10^6^, 10^5^, 10^4^, and 10^3^ copy/μl, which were used as standards for quantification.

**Table 1:** Distribution of repressor element-1 silencing transcription factor (REST)-positive cells in the midbrain of Nile tilapia, *Oreochromis niloticus*.

Brain region	Abbreviation	*REST*-positive cells
Mesencephalon (midbrain)		
Optic tectum	OT	++++
**Semicircular torus**		
Semicircular torus (layer 2)	TS2	+++
Semicircular torus (layer 3)	TS3	+
**Tegmentum**		
Dorsal tegmental nucleus	DTN	+
Rostral tegmental nucleus	RT	++
Perilemniscal nucleus	pL	+
Oculomotor nucleus	NIII	+++
Medial nucleus	MR	++
Superior nucleus	SR	++

*^++++^Very high; ^+++^high; ^++^moderate; ^+^low*.

### *In situ* Hybridization (ISH)

#### RNA Probe Synthesis

Primer design was conducted for RNA probe synthesis as complementary to nucleotides 846-1327 of Nile tilapia *REST* mRNA from whole-brain cDNA (Supplementary Table 1). pGEM-T Easy Vector was used to clone the Nile tilapia *REST* gene fragment (482 bp). The fragment sequence was by 3310 Genetic Analyzer. Antisense and sense *REST* RNA probes were prepared from SalI and NcoI linearized pGEM T-Easy/*REST* plasmid by T7 and SP6 RNA polymerases, respectively. DIG-RNA labeling mix (Roche Diagnostic, Risch-Rotkreuz, Switzerland) was used for *REST* riboprobe preparation. The transcription mixture (10 μl) consisted of linearized plasmid (5 μl), T7 or SP6 polymerase (0.5 μl), transcription-optimized 5× buffer (2 μl), 100 mM DTT (1 μl), DIG RNA labeling mix (1 μl), and RNase inhibitor (0.5 μl) which was incubated at 37°C for 2 h. Labeled probes were purified twice by adding 100% ethanol and was dissolved in 30 μl of ultrapure Milli-Q.

#### ISH Using DIG-Labeled RNA Probe

Adult male tilapia fish brain tissues were collected and fixed with 4% paraformaldehyde for 6 h at 4°C, followed by cryoprotection with 20% sucrose overnight. The brain samples were embedded in a frozen section compound and coronally sectioned in 15-μm slices using a cryostat. Silane-coated slides (Muto Pure Chemicals, Tokyo, Japan) were used for mounting sections. Approximately 160–170 coronal sections (15 μm) were cut, and alternate sections were collected onto a different slide from −0.2 Bregma point (Soga et al., 2012), including the whole midbrain area.

Collected sections were permeabilized with 0.2 M HCl at RT for 10 min, followed by digestion with proteinase K (1 μg/ml) at 37°C for 15 min. Tissues were hybridized with DIG-labeled probes (0.2 ng/1 ml of hybridization buffer) and incubated in sealed humidity chambers overnight at 50°C in the dark for hybridization. Next, slides were washed for 20 min in 2× SSC at RT, two times of 2× SSC wash for 20 min at 55°C, and two times of 0.1× SSC wash for 20 min at 55°C. A series of SSC washes were followed by blocking with 2% normal sheep serum (NSS) before incubating slides with alkaline phosphatase (AP)-conjugated anti-DIG antibody (1:500 dilution; Roche Diagnostic, Risch-Rotkreuz, Switzerland) to detect DIG-labeled RNA probes. Color change reaction was performed using 4-nitro blue tetrazolium and 5-bromo-4-chloro-3-indolyl-phosphate (NBT/BCIP; Roche Diagnostics, Risch-Rotkreuz, Switzerland). Color formation was stopped in tap water.

A Nikon Eclipse 50i light microscope (Nikon, Tokyo, Japan) was used to confirm ISH signals. Magnified images from each section were taken by a digital cool CCD camera (D5-F1; Nikon), and the whole-view section images were taken using Zeiss MIRAX MIDI Slide Scanner (Carl Zeiss, Oberkochen, Germany) with a Panasonic scanner Nikon-30 Confocal microscope (C1si, Nikon Instruments, Tokyo, Japan). The number of *REST* mRNA-positive cells was subjectively determined as follows: ++++ (very high), +++ (high), ++ (moderate), + (low) in each area.

### Double ISH and Immunocytochemistry (ICC)

Adult male tilapia fish was anesthetized with 0.02% benzocaine, and the brain tissues were prepared according to the procedure above. ICC was performed immediately, followed by the ISH process. After SSC washing steps and blocking with 2% NSS in the ISH process, brain sections were incubated with horseradish peroxidase (HRP)-conjugated anti-DIG antibody (1:500, Roche Diagnostic, Risch-Rotkreuz, Switzerland). The primary antibody was detected with Alexa Fluor 594-Tyramide conjugate (1:100 dilution, T20935, Life Technologies, CA, USA) at RT for 30 min. Then, the brain sections were incubated with three antibodies: primary monoclonal mouse anti-HuC/D antibody (1:500, A21271, RRID: AB_221448, Thermo Fisher Scientific, Waltham, MA, USA), primary polyclonal rabbit anti-Glial Fibrillary Acidic Protein (GFAP) antibody (1:500, Z0334, RRID: AB_10013382, Dako, Glostrup, Denmark), or primary polyclonal rabbit anit-5-HT antibody (1:1,000, 20,080, RRID: AB_572263, Immunostar, Hudson, WI, USA). The solution was prepared in 0.01 M PBS (pH 7.0) with 0.5% Triton-X and 2% normal goat serum and incubated for 24 h (HuC/D, GFAP antibody) or 48 h (anti-5-HT antibody) in sealed humidity chambers at 4°C. They were further incubated for 30 min at RT with biotinylated anti-mouse IgG (1:200, PK-6102, RRID: AB_2336821, Vectastain ABC Elite Kit, Vector Laboratories, CA, USA), or anti-rabbit IgG (1:200, PK-6101, RRID: AB_2336820, Vectastain ABC Elite Kit, Vector Laboratories, CA, USA) and then incubated with avidin-biotin-HRP reagent (1:50, Vectastain ABC Elite Kit, Vector Laboratories) for 45 min at RT. Sections were visualized by streptavidin-conjugated Alexa Fluor 488 (1:500, S32354, RRID: AB_2315383, Invitrogen Corporation, CA, USA).

A fluorescence microscope (ECLIPS 90i, Nikon, Tokyo, Japan) equipped with a NIS-Element 3.0 Software was used to confirm double-ISH and ICC signals and take all images. Co-localization was examined using a confocal microscope (Nikon-30, ECRIPS C1si, Nikon, Tokyo, Japan). The images were scanned with XYZ-directions, and 3D confocal data was carefully observed to confirm fluorescence signals’ overlap. The percentage of *REST*-positive 5-HT neurons in PVO, PPd, PPv, DRN, and MRN was determined using scanned images by a confocal microscope. The area of interest for each subject was defined as the area contained at 5-HT-immunoreactive cells. Cell counting of 5-HT neurons was performed manually based on observation from the selected images, which have a maxima 5-HT-immunoreactive cell number per section. A 5-HT neuron (green fluorescence) and REST staining (red fluorescence) in the 5-HT neuron were counted as positive if the cell bodies with well-defined borders contained detectable fluorescence staining. The total number of 5-HT neurons and the percentage of *REST*-positive 5-HT neurons were calculated. Nomenclature for the brain area was adopted from Ogawa et al. (2016) and Cham et al. (2017, 2018).

### Data Analysis

The variations in the expression levels of *REST* genes were observed using the derived boxplot R-package^1^. Data analysis for real-time PCR was done by IBM SPSS Statistic version 23 (IBM, New York, NY, USA). One-way ANOVA was used to compare differences between different brain areas. Data are presented as means ± SEM. *p* < 0.05 was considered statistically different.

## Results

### REST Gene Expression Levels in the Intact Brain

**Figure 1:**
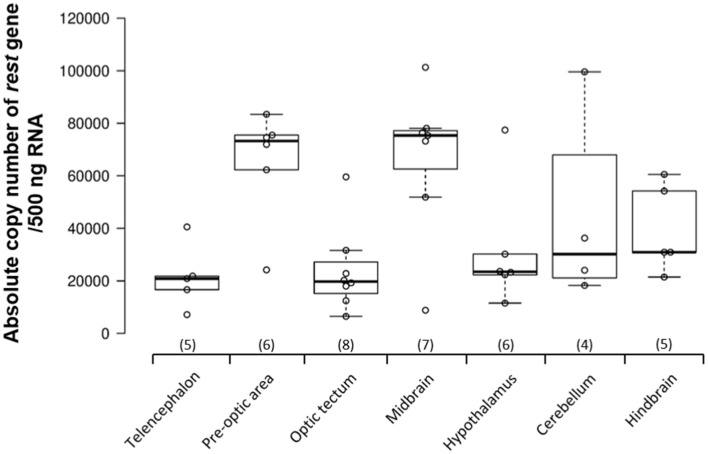
Repressor element-1 silencing transcription factor (*REST*) gene expression in the brain of adult male Nile tilapia. *REST* gene expression levels were examined in seven different brain areas, including the telencephalon, pre-optic area, optic tectum, midbrain, hypothalamus, cerebellum, and hindbrain in adult male Nile tilapia. Brain areas collected during microdissection (telencephalon, *n* = 5; pre-optic area, *n* = 6; optic tectum, *n* = 8; midbrain, *n* = 7; hypothalamus, *n* = 6; cerebellum, *n* = 5; and hindbrain, *n* = 5) for *REST* gene expression study. The number inside each column shows the sample size in the region. All data are shown as mean ± SEM.

*REST* gene expression was examined in seven brain regions of the Nile tilapia: telencephalon, pre-optic area, optic tectum, midbrain, hypothalamus, cerebellum, and hindbrain. All the brain regions showed over 20,000 copy numbers of *REST mRNA* (telencephalon: 21,406.3 ± 5,436.4; optic tectum: 23,808.3 ± 5,726.4; pre-optic area: 65,319.3 ± 8,680.0; hypothalamus: 31,413.2 ± 9,528.0; cerebellum: 44,558.9 ± 18,721.1; hindbrain: 39,611.0 ± 7,514.4). REST mRNA levels were the highest in the midbrain compared to other brain regions (midbrain: 66,429.0 ± 11,028.2; Figure 1).

### Localization of REST Positive Cells in the Midbrain

**Figure 2:**
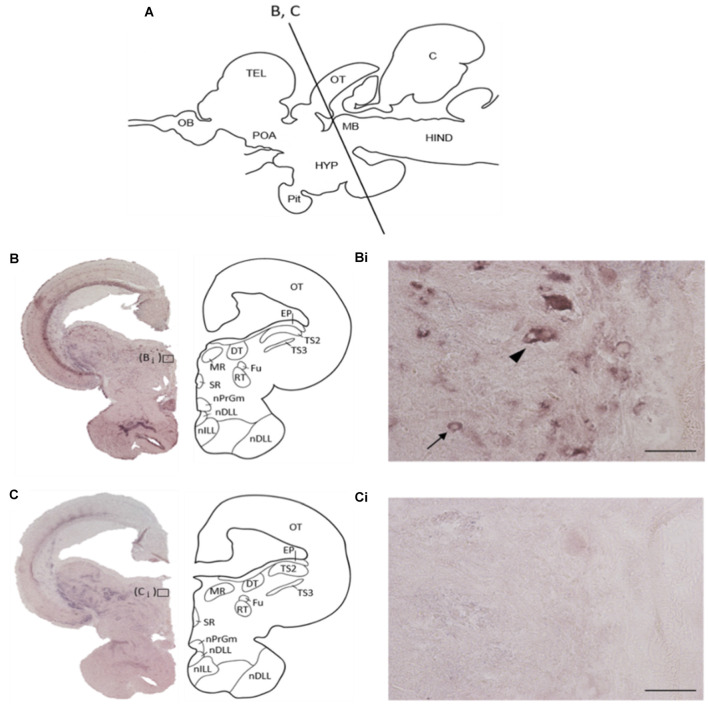
Specificity of tilapia *REST* RNA probe in the midbrain. **(A)** Schematic diagram of midbrain coronal section in Nile tilapia brain with approximate brain regions. **(B)** *In situ* hybridization with anti-sense *REST* mRNA probe, showing a representative whole midbrain coronal section with approximate brain regions. **(Bi)** The higher magnification of **(B)**. Arrow indicates *REST*-positive cells, and arrowhead may show motor neuron from the morphological feature. **(C)** *In situ* hybridization with a sense *REST* mRNA probe, showing a representative whole midbrain coronal section with approximate brain regions. **(Ci)** The higher magnification of panel **(C)**. OT, optic tectum; EP, epiphysis; TS2, layer 2 of semicircular torus; TS3, layer 3 of semicircular torus; DT, dorsal terminal nucleus of accessory optic tract; MR, medial nucleus; SR, superior nucleus; Fu, nucleus of stria terminalis; RT, rostral tegmental nucleus; nPrGm, medial subdivision of the preglomerular nucleus; nDLL, diffuse nucleus of the lateral lobe; nILL, intermediate nucleus of the lateral lobe. Scale bars: **(Bi**,**Ci)**; 50 μm.

**Figure 3:**
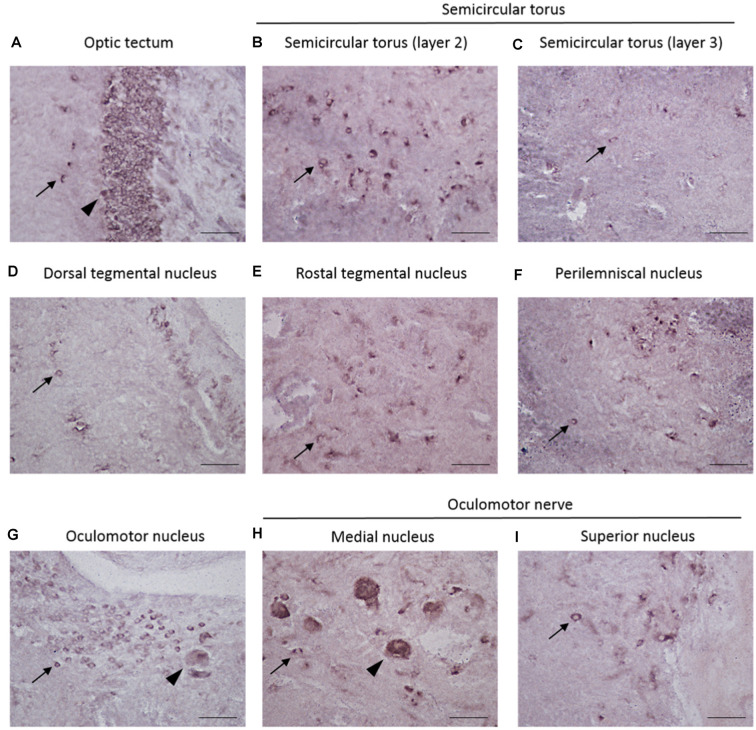
Localization of *REST*-positive cells in the midbrain of male Nile tilapia. **(A–I)** High-magnification photomicrographs of *REST*-positive cells in midbrain regions. Arrow in panels **(A–I)** Indicates *REST*-positive cells. **(A)** The optic tectum. **(B)** Layer 2 of semicircular torus. **(C)** Layer 3 of semicircular torus. **(D)** Dorsal tegmental nucleus. **(E)** Rostral tegmental nucleus. **(F)** Perilemniscal nucleus. **(G)** Oculomotor nucleus. **(H)** Medial nucleus in oculomotor nerve. **(I)** Supervisor nucleus in the oculomotor nerve. Arrowhead in panel **(A)** shows very dense positive cells at the stratum periventriculare (SPV) in the optic tectum, and other arrowheads in panel **(G)**, panel **(H)** may show motor neuron judging from the morphological feature. Scale bars: **(A–I)** 50 μm.

The localization of *REST*-expressing cells was observed in the midbrain by ISH (Figure 2). The tilapia-specific anti-sense *REST* RNA probe showed clear cell staining in the midbrain, whereas the sense probe did not show hybridization signals (Figures 2Bi,Ci). Furthermore, strong stainings were observed in the optic tectum (OT) mainly located in the stratum periventriculare (SPV), layer 2 of the semicircular torus (TS2), layer 3 of the semicircular torus (TS3), and oculomotor nucleus (NIII). *REST*-positive cells were also found in the dorsal (DTN) and rostral tegmental nucleus (RT). Besides, *REST* signals were seen in the perilemniscal nucleus (pL) and medial (MR) and superior nuclei of the nervus oculomotorius (SR; Figure 3). The size of *REST*-positive cells was 10–20 μm in diameter. Some very dense bigger-size cells (more than 30 μm in diameter) may be motor neurons judging from the morphological feature, at the SPV area in the OT (Figure 3A) and in the NIII (Figure 3G) and medial nucleus (Figure 3H). The distribution of *REST*-positive cells and the staining density in the midbrain is summarized in Table 1.

### Double Labeling of REST With Neuron–Astrocyte–Cell Markers

**Figure 4:**
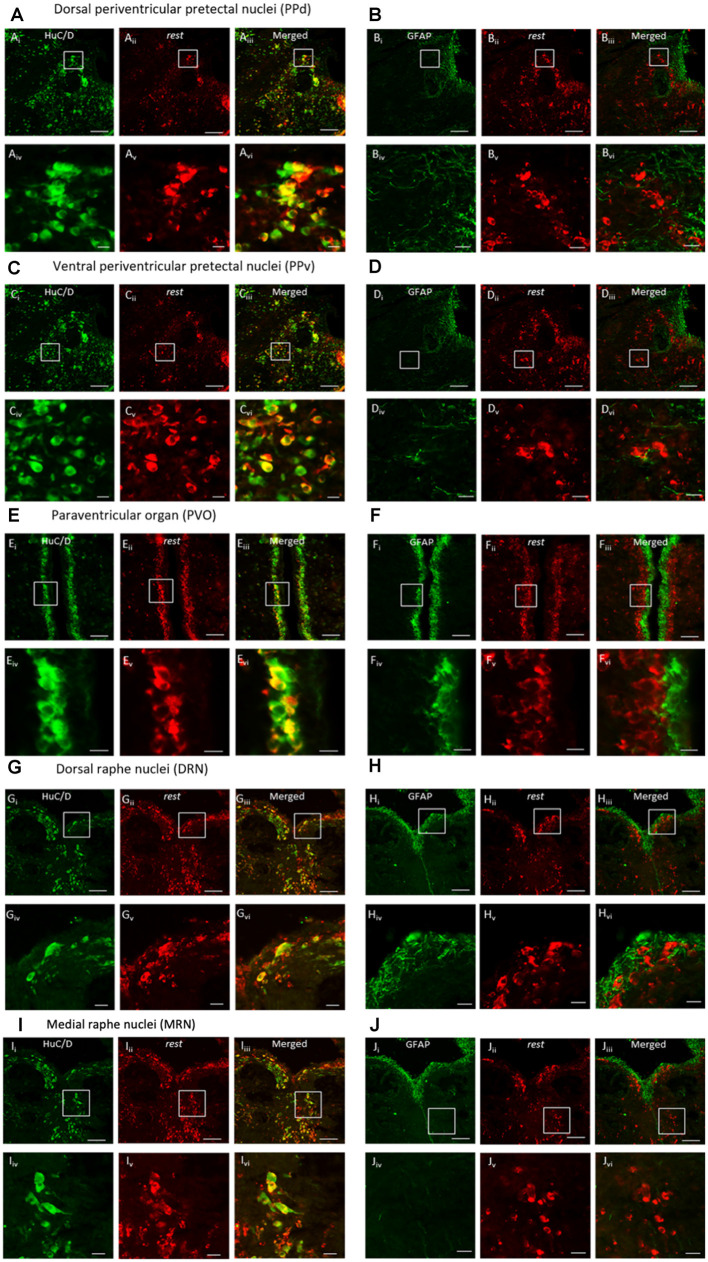
Double-*in situ* hybridization and immunocytochemistry of *REST* and neuronal marker HuC/D or astrocyte marker GFAP. Co-localization was observed in the **(A,B)** dorsal periventricular pretectal nucleus, **(C,D)** ventral periventricular pretectal nucleus, **(E,F)** paraventricular organ nucleus, **(G,H)** dorsal raphe nucleus, and **(I,J)** medial raphe nucleus. Neuronal marker HuC/D (**Ai, Ci, Ei, Gi, Ii**; green), *REST*-positive cell (**Aii, Cii, Eii, Gii, Iii**; red), and merged image **(Aiii, Ciii, Eiii, Giii, Iiii)**. Glial marker GFAP (**Bi, Di, Fi, Hi, Ji**; green), *REST*-positive cells (**Bii, Dii, Eii, Hii, Jii**; red), and merged image **(Biii, Diii, Eiii, Hiii, Jiii)**. **(Aiv–Jiv)** The higher magnification of **(Ai–Ji)**. **(Av–Jv)** The higher magnification of **(Aii–Jii)**. **(Avi–Jvi)** The higher magnification of **(Aiii–Jiii)**. Scale bars: **(Ai–iii)–(Di–iii)**, **(Gi–iii)–(Ji–iii)** 100 μm; **(Ei–iii)–(Fi–iii)** 50 μm; **(Aiv–vi)–(Div–vi)**, **(Giv–vi)–(Jiv–vi)** 20 μm, **(Eiv–vi)–(Fiv–vi)** 10 μm.

Double-ISH and ICC showed clear expression of *REST* co-localized with neuronal marker HuC/D in the dorsal and ventral periventricular pretectal nucleus (PPd and PPv), paraventricular organ nucleus (PVO), and dorsal raphe nucleus (DRN; Figure 4). The *REST* gene, although not all, was expressed in neuronal cells in the PPd, PPv, PVO, and DRN (Figures 4A,C,E,G). Correspondingly, double-ISH and ICC for *REST* with astrocyte marker GFAP showed the absence of *REST* mRNA in astroglial cells in the PPd, PPv, PVO, and DRN (Figures 4B,D,F,H).

### Double Labeling of REST and 5-HT-Positive Cells

**Figure 5:**
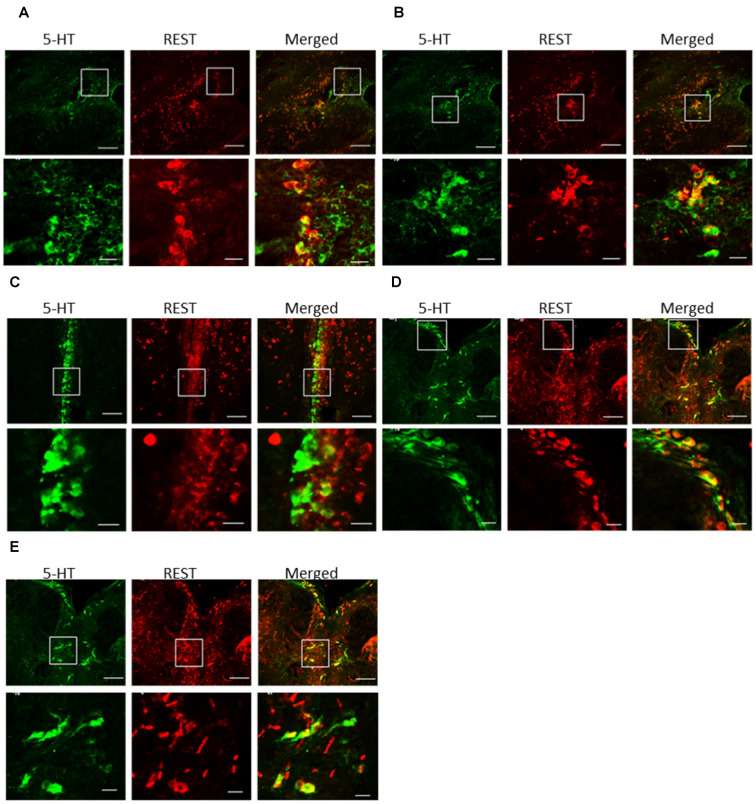
Double-*in situ* hybridization and immunocytochemistry of *REST* and 5-HT immunoreactive cells. Co-localization was examined in the **(A)** dorsal periventricular pretectal nucleus, **(B)** ventral periventricular pretectal nucleus, **(C)** paraventricular organ nucleus, **(D)** dorsal raphe nucleus, and **(E)** medial raphe nucleus. 5-HT immunoreactive cell (green), *REST*-positive cell (red), and merged image (yellow). The scare in low-magnification images (higher panels) enlarges in high magnification images (lower panels). *REST*-positive 5-HT neurons were indicated by arrows. Scale bars: low-magnification images (higher panels) in **(A–D)** 100 μm;**(E)** 50 μm, high-magnification images (lower panels) in **(A–D)** 20 μm; **(E)** 10 μm.

Double staining with ISH and immunofluorescence showed co-localization of *REST* mRNA in 5-HT cells in the periventricular region (PPd and PPv), paraventricular organ (PVO), and raphe nucleus (DRN and MRN; Figure 5). Not all 5-HT-positive neurons were *REST* positive in each nucleus. Our preliminary data of cell counts showed that the highest percentage of *REST*-positive 5-HT neurons in the raphe region and the lowest percentage of *REST*-positive 5-HT neurons were present in the PVO (24.8 ± 5.5% in PVO, 57.6 ± 3.3% in the PPd, 64.0 ± 8.2% in PPv, 67.4 ± 6.9% in the DRN, and 76.1 ± 6.9% in the MRN).

## Discussion

### Distribution of REST-Positive Cells in the Midbrain

Real-time quantitative analysis showed *REST* mRNA in all brain regions of the Nile tilapia. In particular, the midbrain expression level was high; therefore, we focused on REST expression in the midbrain in this study. The *REST*-positive cells were seen throughout the midbrain area. A large number of *REST*-positive cells are specifically observed in the optic tectum (TeO), layer 2 of the semicircular torus (TS2), and oculomotor nucleus (NIII). These three brain areas are critical processing centers for sensory information. In mammals, the corresponding homologous structures of TeO and TS are known as the superior colliculus and inferior colliculus, respectively (Meek, 1998). The superior colliculus receives visual inputs in the superficial layers, while the inferior colliculus is involved in auditory–somatosensory interaction.

On the other hand, NIII regulates oculomotor nerve innervation of eye muscles (Ruchalski and Hathout, 2012). In fish, TeO is one of the largest brain structures with various motor functions such as swimming and avoidance behavior (Nevin et al., 2010; Mishra and Devi, 2014). It has been shown that electrical stimulation given to superior colliculus or inferior colliculus of rats induces fear- and anxiety-like behaviors (Melo et al., 1992; Coimbra and Brandão, 1997). There is a contribution of the tectal division of the midbrain (e.g., superior and inferior colliculus) and the periaqueductal gray in anxiety-like behavior (Brandão et al., 2003; Taylor et al., 2019). Furthermore, this midbrain circuitry is related to defensive behavior (Tovote et al., 2016) and locomotion speed (Caggiano et al., 2018). Thus, the presence of *REST* mRNA and *REST*-positive cells throughout the midbrain suggest that REST could be involved in the modulation of various functions such as emotional-, anxiety-, and depressive-like behaviors and sensory and motor activities associated with some of the midbrain nuclei. The expression and activities of REST have been shown to be different at specific brain regions in normal and pathological conditions (Kaneko et al., 2014; Lu et al., 2014; Hwang and Zukin, 2018). Besides, the cellular function-regulated REST is neuronal-type-dependent (Hwang and Zukin, 2018).

Besides, the midbrain and hindbrain raphe nuclei are well-known 5-HT neuronal-containing regions in vertebrate species (Adell et al., 2002; Prasad et al., 2015). In teleost, raphe 5-HT neurons project their fibers to TeO, TS2, and NIII brain regions (Kaslin and Panula, 2001), which are highly dense *REST* areas, suggesting that REST may regulate 5-HT system-related responses.

### Expression of REST in Glial Cells and Neurons

REST expression has been reported in glial cells, including astrocytes, microglia, oligodendrocytes (Abrajano et al., 2009a; Prada et al., 2011), in glioma (Ren et al., 2015; Li et al., 2017). The expression of REST in glial cells might depend on the physiological or developmental stage or a pathological condition of the animal, such as glioma in the brain. However, more studies on the cellular functions of REST in glial cells are required.

In our study, REST was co-localized in cells expressing HuC/D but not in cells expressing GFAP, which shows that REST is exclusively expressed in neurons and not in astrocytes in the brain of tilapia. In mammals, REST expression has been reported in neurons in specific brain areas (Lu et al., 2014; Schiffer et al., 2014).

Emerging evidence has linked REST expression in specific neurons to cellular functions (Baldelli and Meldolesi, 2015) Recent studies have shown dopaminergic neurons to express REST, which activates the expression of dopamine-synthesizing enzyme tyrosine hydroxylase and thereby protects neurons against cell toxicity (Kawamura et al., 2019; Pajarillo et al., 2020). Therefore, we speculate that the expression of REST in the midbrain, in our study, might be co-expressed in some subpopulation of dopaminergic neurons. In addition to the midbrain, REST expression in the pre-optic area might be co-localized in gonadotropin hormone-releasing hormone (GnRH)-synthesizing neurons.

Indeed, a recent study has shown REST co-expressed in GnRH neurons, which increases the functional expression of calcium channels and reduces their migratory potential (Antoniotti et al., 2016). The chemical nature of REST-positive neurons in the pre-optic and midbrain areas needs to be identified. This would help to elucidate the cellular function of REST in these neurons.

### Interaction Between REST and 5-HT Neurons

The expression of *REST* was seen in many nuclei throughout the midbrain and hindbrain areas as well as co-expression in 5-HT neuronal populations (PPd, PPv, PVO, DRN, and MRN). These 5-HT nuclei are widely interconnected with rostral and caudal brain regions to control neuroendocrine and behavioral activities. For instance, in the rainbow trout, the midbrain and the hindbrain are important targets for antidepressant drugs, shown by a region-specific drug impact on neurotransmitter levels of 5-HT, dopamine, and norepinephrine (Melnyk-Lamont et al., 2014). It implies that neuronal *REST* in these brain areas could alter the neurotransmitter systems, including 5-HT in response to stress.

Several 5-HT-related genes have transcriptional regulator REST-binding RE1 sites. REST-dependent transcriptional repression of molecules related to 5-HT synthesis and 5-HT reuptake at the synapses suggest the role of REST in 5-HT synthesis and release (Patel et al., 2007; Albert et al., 2011; Nawa et al., 2017). Numerous reports indicate a close link between a change in molecules related to 5-HT and a reduction of 5-HT in the brain (Gardner et al., 2009; Mineur et al., 2015; Soga et al., 2020), which may be mediated by REST. A previous report shows that approximately 2,000 putative REST target genes (Mortazavi et al., 2006) suggest that novel REST target genes could be involved in 5-HT neuronal functions and regulation.

In the present study, a higher percentage of REST-positive 5-HT neurons were seen in the DRN and MRN and a lower percentage in the PPd, PPv, and PVO areas. In mammals, 5-HT neurons in DRN and MRN can be differentiated by their location, morphology, and functional properties (Abrams et al., 2004). These subnuclei possess different electrophysiological characteristics and excitatory responses to stressful stimuli (Abrams et al., 2004; Beck et al., 2004). For instance, increased cFos expression, a marker for neuronal activation, is found in 5-HT neurons of the DRN, but not in MRN during stress (Cooper et al., 2009). This suggests that different mechanisms might regulate 5-HT neurons of the DRN and MRN in response to stress. TPH2 promoter activity and 5-HT 1A expression are modulated by REST (Lemonde et al., 2004; Gentile et al., 2012), which suggests that the expression of REST in different subpopulations of 5-HT neurons may be involved in different functions in each population such as 5-HT biosynthesis or autoregulation. Furthermore, a recent in-silico screening study showed that antidepressant and antipsychotic drugs interact with the REST-binding site of the mSin3B PAH1 domain (Kurita et al., 2018), raising the possibility of REST activity in a different 5-HT neuronal population having different pharmacological sensitivity.

Efferent neuronal connections projecting from the PPd/PPv region to the optic tectum have been shown in zebrafish (Yáñez et al., 2018). Activation of the superior colliculus, a homologous structure of the optic tectum in mammals, can induce fear- and anxiety-like behaviors (Melo et al., 1992; Coimbra and Brandão, 1997), and *REST* is highly expressed in the optic tectum. These indicate that REST may be involved in stress-coping mechanisms via neuronal connections between PPd/PPv 5-HT neurons and the optic tectum. In mammals, the PVO region has not been formally recognized but several studies suggest its presence in the dorsomedial hypothalamus (DMH) of rodents (Lowry et al., 1996). It has been shown that stress-induced CRH and corticosterone, which is a glucocorticoid hormone, stimulate 5-HT and 5-HIA_A_ accumulation in the DMH (Lowry et al., 2001). Thus, REST expressed in 5-HT neurons of the PVO might have an important role in HPA axis-related stress response. In this study, the percentage of REST-positive 5-HT cells in each neuronal population is 25–75%. This means that some 5-HT neurons are regulated by REST-related signaling in each 5-HT neuronal population, but other 5-HT neurons may be under a different control. REST expression is regulated by several physiological factors such as age, gender, stress, and the endocrine system in different brain regions (Soga et al., 2020). Besides, the expression and activities of REST have been shown to be different at specific brain regions in normal and pathological conditions (Kaneko et al., 2014; Lu et al., 2014; Hwang and Zukin, 2018). Furthermore, the cellular function regulated by REST is neuronal-type-dependent (Hwang and Zukin, 2018). The relationship between the difference of the REST expression pattern in 5-HT neurons and the physiological function may be needed for further study.

## Conclusion

Our results show the expression of *REST* gene in many nuclei throughout the midbrain and hindbrain and co-expression in 5-HT neuronal populations (PPd, PPv, PVO, DRN, and MRN). *REST* expression in different 5-HT neuronal populations might have other functions in the regulation of 5-HT and could be a potential new therapy against 5-HT dysfunction-related disorders.

## Data Availability Statement

The raw data supporting the conclusions of this article will be made available by the authors, without undue reservation.

## Ethics Statement

The animal study was reviewed and approved and all animal experimental procedures were approved and conducted according to the guidelines of Monash University Animal Ethics Committee, AEC (approval; MARP/2015/109, MARP/2015/180).

## Author Contributions

TS and IP designed all experiments and edited the manuscript. TS and SN conducted all experiments, wrote the main manuscript and prepared all figures and table. SN and TS analyzed all data together. All authors reviewed the manuscript. All authors contributed to the article and approved the submitted version.

## Conflict of Interest

The authors declare that the research was conducted in the absence of any commercial or financial relationships that could be construed as a potential conflict of interest.

## Footnotes

^1^http://boxplot.tyerslab.com
