# Supplementary material for: Expression of Repressor Element 1 Silencing Transcription Factor (REST) in Serotonin Neurons in the Adult Male Nile Tilapia (Oreochromis niloticus)
Source: Front Neuroanat. 2021 Mar 11;14:599540. doi: 10.3389/fnana.2020.599540 (PMC7990894; doi:10.3389/fnana.2020.599540)
Supplement: Supplementary file 1 [file Table_1.pdf]

Supplemental table1 : Primer sets for real-time PCR and *in situ* hybridization.

Gene names	Primers	Nucleotide sequence	Product length (bp)	Accession no.
rest	F	5'-GTGGATGTGACGGTGAGAAGGA-3'	92	XM_003443432.4
	R	5'-ACCATACCTGATCACACTCTCGT-3'		
rest (probe)	F	5'-CACCACCGTCAGTCAGTACCA-3'	482	XM_003443432.4
	R	5'-TGCAGCCAGAGTGCCTAGACTT-3'		
β-actin	F	5'-CACCGTGCTGTCTGGAGGTA-3'	120	KJ_126772.1
	R	5'-TTACGCTCAGGTGGGGCAA-3'		
